# A comparison of species specific sensitivities to changing light and carbonate chemistry in calcifying marine phytoplankton

**DOI:** 10.1038/s41598-019-38661-0

**Published:** 2019-02-21

**Authors:** Natasha A. Gafar, Bradley D. Eyre, Kai G. Schulz

**Affiliations:** Centre for Coastal Biogeochemistry, School of Environment Science and Engineering, Southern Cross University, Lismore, NSW 2480 Australia

## Abstract

Coccolithophores are unicellular marine phytoplankton and important contributors to global carbon cycling. Most work on coccolithophore sensitivity to climate change has been on the small, abundant bloom-forming species *Emiliania huxleyi* and *Gephyrocapsa oceanica*. However, large coccolithophore species can be major contributors to coccolithophore community production even in low abundances. Here we fit an analytical equation, accounting for simultaneous changes in CO_2_ and light intensity, to rates of photosynthesis, calcification and growth in *Scyphosphaera apsteinii*. Comparison of responses to *G. oceanica* and *E. huxleyi* revealed *S. apsteinii* is a low-light adapted species and, in contrast, becomes more sensitive to changing environmental conditions when exposed to unfavourable CO_2_ or light. Additionally, all three species decreased their light requirement for optimal growth as CO_2_ levels increased. Our analysis suggests that this is driven by a drop in maximum rates and, in *G. oceanica*, increased substrate uptake efficiency. Increasing light intensity resulted in a higher proportion of muroliths (plate-shaped) to lopadoliths (vase shaped) and liths became richer in calcium carbonate as calcification rates increased. Light and CO_2_ driven changes in response sensitivity and maximum rates are likely to considerably alter coccolithophore community structure and productivity under future climate conditions.

## Introduction

Coccolithophores are a group of phytoplankton (division Haptophyta, class Prymnesiophyceae), which produce both particulate organic carbon through photosynthesis and particulate inorganic carbon through calcification^1,2^. They are found in almost all ocean ecosystems from the equator to sub-polar latitudes, from nutrient rich upwelling zones to nutrient poor oligotrophic regions and from surface waters to over 100 m in depth^3–7^. Within ocean ecosystems coccolithophores play an important role in the production and cycling of carbon contributing up to 20% to total organic carbon fixation^8,9^ and up to 50% of calcium carbonate flux to marine sediments^10–12^. Because of this, coccolithophores have become amongst the most intensively studied organisms in terms of the effects of ocean acidification in particular and climate change in general (e.g.^13–16^). Recent work, suggests that coccolithophores have a common over-arching response to changes in carbonate chemistry^16–18^. However, these responses vary between species, strains and additionally can be modulated by changes in light intensity and temperature^16,19,20^.

**Figure 1:**
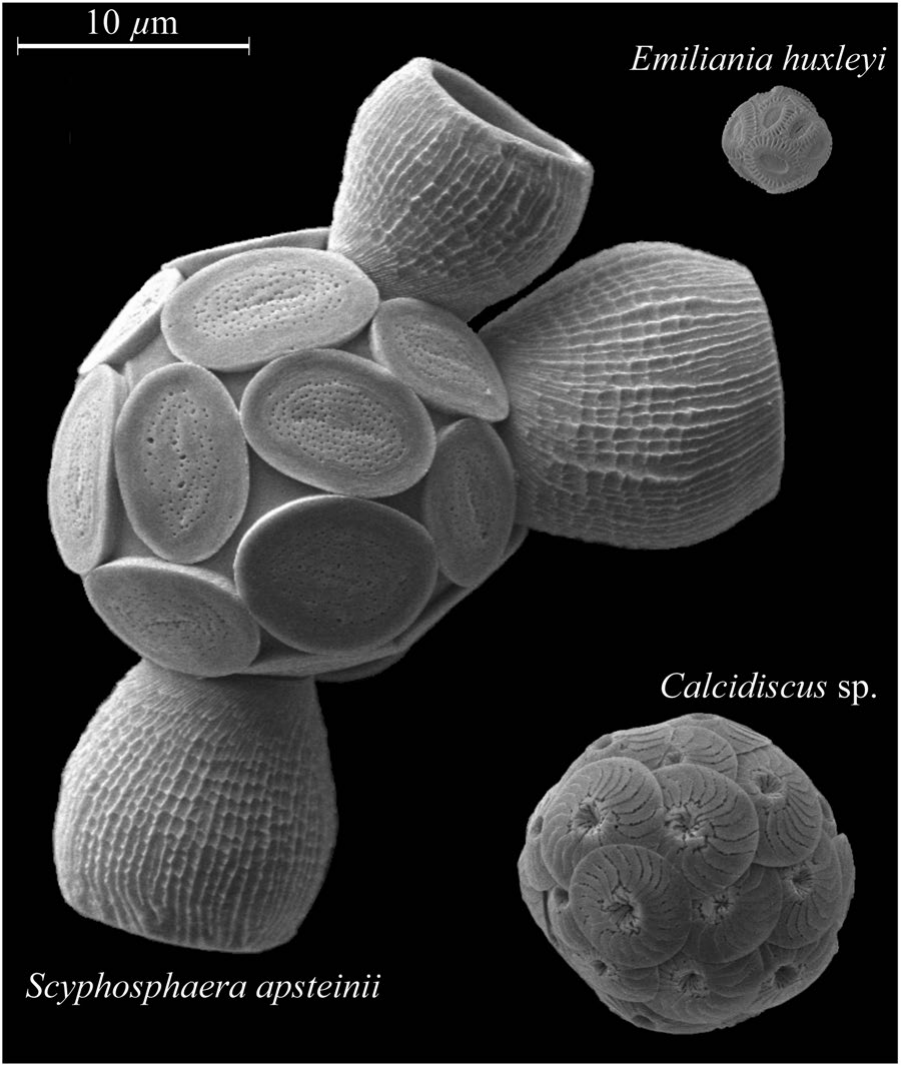
Scanning electron microscope images of a *E. huxleyi*, *S. apsteinii* and a *Coccolithus* species showcasing size and cellular calcium carbonate quota differences between species. Species shown were either isolated or cultured by the authors.

Of approximately 200 extant species of coccolithophores (e.g.^21^), the majority of research into coccolithophore responses to climate change come from only a few species such as *Emiliania huxleyi*, *Gephyrocapsa oceanica*, *Calcidiscus leptoporus*, and *Coccolithus pelagicus spp. braarudii*. Of these most studies have been on *E. huxleyi* which is considered a keystone species due to its ability to form massive blooms^22^, its dominance within the coccolithophore assemblage^3,6,23^, and its almost global distribution^3^ (North and South Atlantic),^22^ (North Atlantic),^6^ (Equatorial and sub-equatorial Pacific),^24^ (East Mediterranean),^25,26^ (Southern Ocean),^27^ (North Sea). While potentially dominant in terms of abundance *E. huxleyi* s contribution to both the organic and inorganic carbon cycle is likely less of importance. Among the coccolithophores *E. huxleyi* is quite small (cell size ∼5 *μ*m) and has a fairly low cellular calcium carbonate (CaCO_3_) content (2.88 to 12.12 pg C cell^−1^)^16,28–30^ (Fig. 1). Meanwhile larger and less abundant species such as *C. pelagicus* (∼16 *μ*m), *C. leptoporus* (∼13.8 *μ*m), *Helicosphaera carteri* or *Scyphosphaera apsteinii* (∼17.6 *μ*m) produce more than 20 times this amount^29,31,32^. Recent research suggests that these larger and more CaCO_3_ rich species may be the dominant contributors to carbon production and export in the coccolithophore assemblage^10,29,33–35^. Despite this, little is known about the effects of climate change on these larger species. As such the aim of this study was to examine the effects of changing carbonate chemistry and light intensity on the large coccolithophore *S. apsteinii*.

## Results

**Figure 2:**
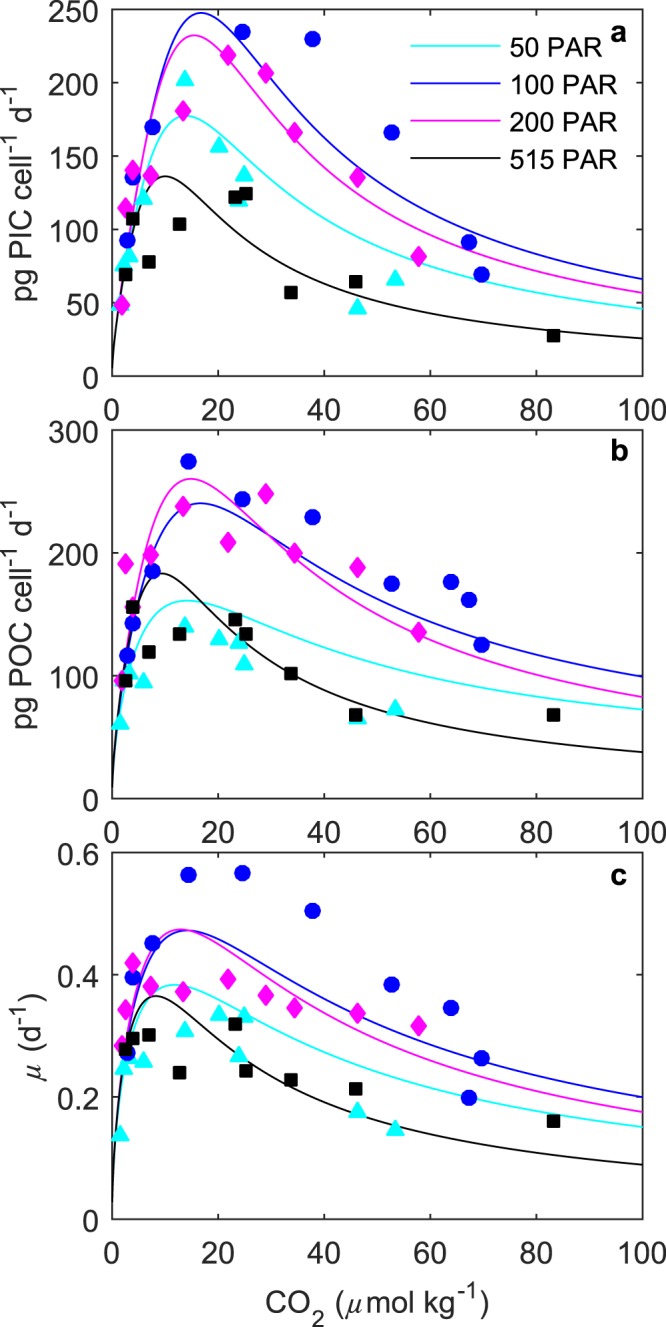
Fitted (solid lines) and measured (symbols) (**a**) particulate inorganic carbon (PIC), (**b**) particulate organic carbon (POC) production, and (**c**) growth rates in response to changes in CO_2_ concentration at six different light intensities using equation 2 and fit coefficients from Table 1. Symbols represent rate measurements at a constant temperature (20 °C) and 50 (Δ), 100 (◯), 200 (◊) and 515 (□) *μ*mol photons m^−2^ s^−1^.

**Figure 3:**
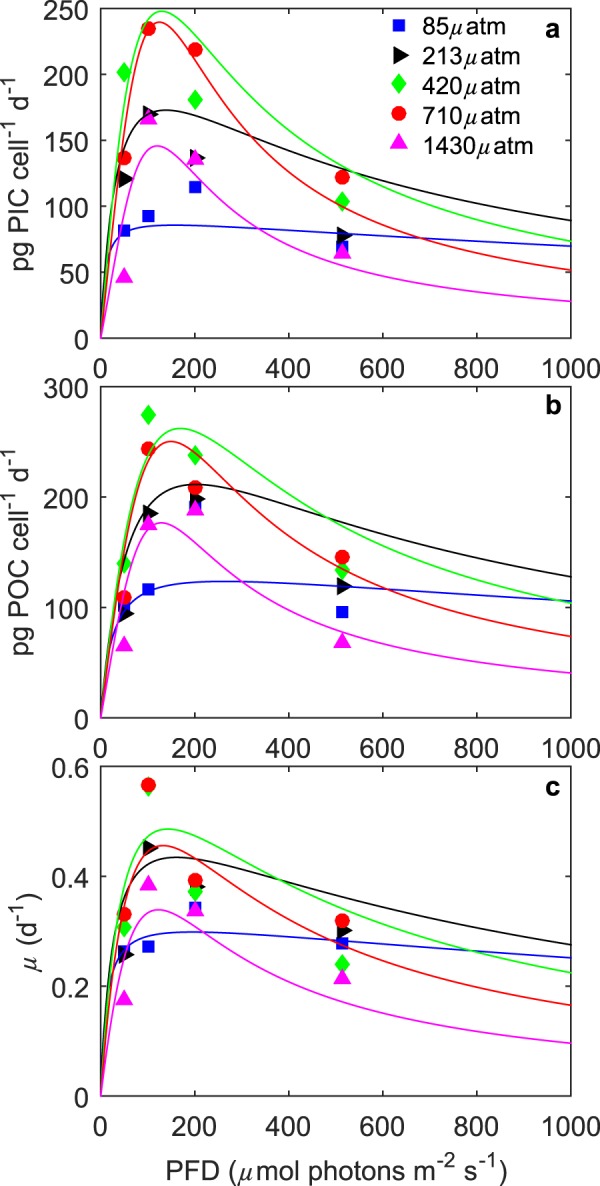
(**a**) Particulate inorganic carbon (PIC) and (**b**) particulate organic carbon (POC) production, and (**c**) growth rates of *S. apsteinii* in response to changes in light intensity at five different *f* CO_2_ levels at a constant temperature (20 °C). Symbols denote measured data at *f* CO_2_ levels of ∼85 (□), ∼213 (⊳), ∼420 (◊), and ∼710 (◯) and ∼1430 (Δ) *μ*atm and lines represent calculated rates using equation 2 and fit coefficients in Table 1.

Growth of *S. apsteinii* cultures was observed between starting conditions of 50–5000 *μ*atm, but not at 7000 *μ*atm. The fit to combined light and carbonate chemistry conditions (Eq. 2) explained up to 75% of the variability in measured response rates across a range of carbonate chemistry (47–2570 *μ*atm) and light (50–515 *μ*mol photons m^−2^ s^−1^) conditions (Table 1). The equation was able to capture the limiting, stimulating and inhibiting effects of changing carbonate chemistry (Fig. 2) and light intensity (Fig. 3) on all physiological rates.

**Table 1:** Fit coefficients (k_1_, k_2_, k_3_, k_4_, k_5_, k_6_), R^2^, p-values, F-values and degrees of freedom obtained from fit equation (2) for calcification (pg C cell^−1^ d^−1^), photosynthetic carbon fixation (pg C cell^−1^ d^−1^) and growth rate (d^−1^) fits to all data. Note that for calcification and photosynthetic carbon fixation rates the unit of k_1_ is pg C cell^−1^ day^−1^ and for growth rates day^−1^.

	Calcification	Photosynthesis	Growth rate
k_1_ (pg C cell^−1^ day^−1^ or day^−1^)	5.77E + 13	−1.18E + 05	−7.57E + 04
k_2_ (*μ*mol photons m^−2^ s^−1^)	2.56E + 18	−1.79E + 10	−1.97E + 12
k_3_ (kg mol^−1^ *μ*mol photons m^−2^ s^−1^)	−1.00E + 03	6.21E + 12	4.78E + 14
k_4_ (mol kg^−1^)	2.29E + 09	−2.81	−569.84
k_5_ (dimensionless)	−1.15E + 12	1.35E + 03	2.12E + 05
k_6_ (kg mol^−1^ *μ*mol photons^−1^ m^2^ s)	7.95E + 16	−1.09E + 08	−2.69E + 10
R^2^	0.7501	0.7012	0.5727
(p-value)	(4.43E-12)	(3.00E-11)	(2.41E-8)
F-value	105.05	86.91	49.74
Degrees of freedom	35	37	37

### Responses to changing carbonate chemistry

Growth, photosynthetic carbon fixation and calcification rates all had an optimum curve response to increasing CO_2_ (Fig. 2, Table S1) at all light intensities. Depending upon CO_2_ level and light intensity, rates varied between 28–234 pg C cell^−1^ d^−1^ for calcification, 60–243 pg C cell^−1^ d^−1^ for photosynthesis and 0.14–0.57 d^−1^ for growth (Table S1). At all light intensities, particulate inorganic to organic carbon ratios PIC:POC ratios increased with CO_2_ to an optimal point before declining with further increases in CO_2_ (Table S1). PIC:POC ratios varied from 0.42 to 1.44 depending upon CO_2_ level and light intensity (Table S1).

Calcification, photosynthetic carbon fixation and growth rates had similar CO_2_ requirements to achieve half saturation $({\rm{K}}\tfrac{1}{2}{}_{{{\rm{CO}}}_{2}}{\rm{s}}{\rm{at}})$ and optimal rates (Table 2). Calcification rates were more sensitive to increasing [H^+^] $({\rm{K}}\tfrac{1}{2}{}_{{{\rm{CO}}}_{2}}{\rm{i}}{\rm{nhib}})$ than photosynthetic carbon fixation or growth rates (Table 2, Fig. 2). Optimum CO_2_ and CO_2_ half-saturation concentrations $({\rm{K}}\tfrac{1}{2}{}_{{{\rm{CO}}}_{2}}{\rm{s}}{\rm{at}})$ changed little (in comparison to the overall CO_2_ response range and uncertainties in the model fit) with increasing light (Table 2). CO_2_ half inhibition concentrations $({\rm{K}}\tfrac{1}{2}{}_{{{\rm{CO}}}_{2}}{\rm{i}}{\rm{nhib}})$ changed little with increasing light for calcification, followed a slight optimum curve response for growth, and decreased with increasing light for photosynthesis (Table 2). Differences in sensitivity to [H^+^] between the three rates decreased with increasing light intensity.

**Table 2:** Optimum CO_2_ concentrations, ${{\rm{CO}}}_{2}{\rm{K}}\tfrac{1}{2}$ concentrations and maximum rates (V_max_) of *S. apsteinii* from fit equation (2) at 50–515 *μ*mol photons m^−2^ s^−1^ and 20 °C using fit coefficients from Table 1.

CO_2_	50 PAR	100 PAR	200 PAR	515 PAR
**CO**_**2**_ **optima (** ***μ*** **mol kg** ^**−1**^ **)**				
Calcification	13.9	16.8	15.5	10.0
Photosynthesis	14.2	16.7	14.8	9.4
Growth rate	11.8	14.1	12.9	8.2
**V**_***max***_ **(pg C cell** ^**−1**^ **d** ^**−1**^ **or d** ^**−1**^ **)**				
Calcification	177.4	247.6	232.3	136.2
Photosynthesis	161.1	240.3	260.2	183.2
Growth rate	0.38	0.47	0.47	0.37
${\bf{K}}\tfrac{1}{2}{}_{{\bf{C}}{{\bf{O}}}_{2}}{\bf{i}}{\bf{nhib}}$ **(** ***μ*** **mol kg** ^**−1**^ **)**				
Calcification	49.9	53.8	49.7	36.7
Photosynthesis	84.1	77.2	59.5	38.3
Growth rate	71.3	79.4	68.0	42.6
${\bf{K}}\tfrac{1}{2}{}_{{\bf{C}}{{\bf{O}}}_{2}}{\bf{s}}{\bf{at}}$ **(** ***μ*** **mol kg** ^**−1**^ **)**				
Calcification	3.2	4.5	4.1	2.2
Photosynthesis	1.9	3.0	3.0	1.8
Growth rate	1.4	1.8	1.8	1.1

### Responses to changing light intensity

Maximum rates for growth, calcification and photosynthetic carbon fixation were observed to increase up to an optimum light intensity before declining with further increases in light (Fig. 3). Calcification rates increased 39% between 50 and 100 *μ*mol photons m^−2^ s^−1^ before declining by 45% at 515 *μ*mol photons m^−2^ s^−1^. Photosynthetic carbon fixation rates increased 62% between 50 and 200 *μ*mol photons m^−2^ s^−1^ and declined by 30% at 515 *μ*mol photons m^−2^ s^−1^. Growth rates increased 24% between 50 and 200 *μ*mol photons m^−2^ s^−1^ and declined by 23% at 515 *μ*mol photons m^−2^ s^−1^ (Table 2). The effect of carbonate chemistry (i.e. the combined change in concentrations of CO_2_, ${{\rm{HCO}}}_{3}^{-}$, ${{\rm{CO}}}_{3}^{2-}$ and H^+^ as a result of rising *f* CO_2_) on metabolic rates was greatest between 100–400 *μ*mol photons m^−2^ s^−1^ and decreased towards more extreme light intensities (Fig. 3).

Increasing *f* CO_2_ resulted in an increase in light half-saturation intensities $({\rm{K}}\tfrac{1}{2}{}_{{\rm{PAR}}}{\rm{s}}{\rm{at}})$, a decrease in optimal light requirements, and an increase in sensitivity to high light $({\rm{K}}\tfrac{1}{2}{}_{{\rm{PAR}}}{\rm{i}}{\rm{nhib}})$ for all rates (Table 3). Calcification reached optimal rates at lower light intensities (120–157 *μ*mol photons m^−2^ s^−1^) than growth (122–196 *μ*mol photons m^−2^ s^−1^) or photosynthesis (128–263 *μ*mol photons m^−2^ s^−1^), though differences between the rates decreased with increasing *f* CO_2_ (Table 3). Inhibiting light levels were lowest for calcification (385–1050 *μ*mol photons m^−2^ s^−1^), higher for photosynthesis (447–1346 *μ*mol photons m^−2^ s^−1^) and highest for growth (521–1524 *μ*mol photons m^−2^ s^−1^).

**Table 3:** Calculated optimum light intensities, maximum rates (V_max_) and light ${\rm{K}}\tfrac{1}{2}$ values of *S. apsteinii* at 20 °C and 85, 213, 420, 710 and 1430 *μ*atm using equation 2 and fit coefficients from Table 1.

Light	85 *μ*atm	213 *μ*atm	420 *μ*atm	710 *μ*atm	1430 *μ*atm
**Optima (** *μ* **mol photons m** ^− **2**^ **s** ^− **1**^ **)**					
Calcification	157	139	130	125	120
Photosynthesis	263	203	169	149	129
Growth rate	196	161	143	133	123
**V**_***max***_ **(pg C cell** ^−1^ **d** ^−1^ **or d** ^−1^ **)**					
Calcification	85.6	172.9	247.9	239.6	145.7
Photosynthesis	123.6	211.4	262.1	250.3	176.6
Growth rate	0.30	0.43	0.49	0.46	0.34
${\bf{K}}\tfrac{1}{2}{}_{{\bf{PAR}}}{\bf{i}}{\bf{nhib}}$ **(** *μ* **mol photons m** ^− **2**^ **s** ^− **1**^ **)**					
Calcification	>2500	1050	548	423	386
Photosynthesis	>2500	1346	754	561	448
Growth rate	>2500	1524	894	667	521
${\bf{K}}\tfrac{1}{2}{}_{{\bf{PAR}}}{\bf{s}}{\bf{at}}$ **(** *μ* **mol photons m** ^− **2**^ **s** ^− **1**^ **)**					
Calcification	7.2	18.3	30.6	36.8	37.4
Photosynthesis	18.4	30.6	38.0	39.7	37.1
Growth rate	9.9	17.0	22.8	26.4	28.8

### Coccolith morphology

**Figure 4:**
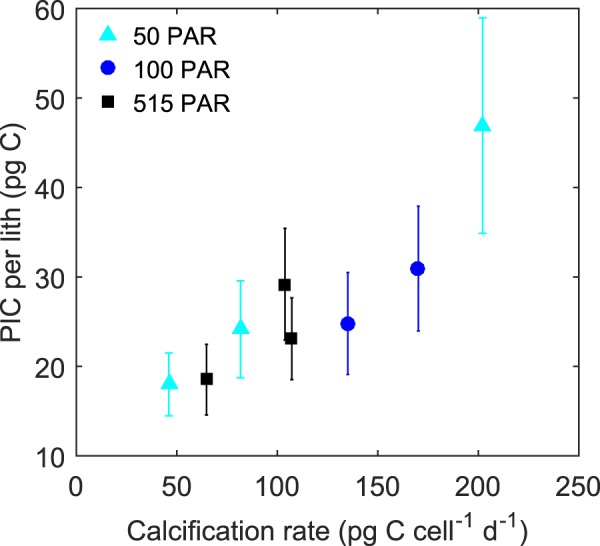
PIC content, by weight, of average *S. apsteinii* coccolith (mix of muroliths and lopadoliths) versus calcification rate under different light and CO_2_ conditions. Error bars represent standard deviation for each treatment condition.

Between the different light and CO_2_ treatments, coccosphere size ranged from 12.0–22.4 *μ*m (average 17.2, Table S2). The number of muroliths per cell varied between five and 23 (average 12.4), while the number of lopadoliths per cell varied between zero and six (average 2.3). The ratio of muroliths to lopadoliths on a cell varied from zero to 22 (average 6.4). The length of muroliths was 6.1–10.9 *μ*m (average 8.3) while the width was 4.0–9.1 *μ*m (average 6.1). The length of lopadoliths varied between 2.8–13.9 *μ*m (average 8.1) and the width 6.1–16.3 *μ*m (average 10.5). The ratio of muroliths to lopadoliths was observed to increase with increasing light at 100 (n = 479 df = 2, Chi-sq = 14.34, p = 7.69E-4) and 400 *μ*atm CO_2_ (n = 357 df = 2, Chi-sq = 11.44, p = 3.28E-3). There was also a significant effect of calcification rate on PIC per lith, with liths containing more PIC in treatments with higher calcification rates (n = 8 R^2^ = 0.80, F = 23.45 p = 2.87E-3, Fig. 4). Other than this, no environmentally significant patterns were observed in any morphological features with changing CO_2_ or light (see supplementary information for discussion on these results).

## Discussion

Increasing CO_2_ concentrations resulted in an optimum curve response in all physiological rates for *S. apsteinii* (Fig. 2). This response pattern has now been observed for multiple coccolithophore species^16,18^, and is most likely driven by the combined effects of physiological rate stimulation by increasing substrate (CO_2_ and ${{\rm{HCO}}}_{3}^{-}$) availability and physiological rate inhibition by increasing [H^+^]^14,17,18,36^.

Larger species generally require more substrate than smaller species to sustain growth. However, larger species have less surface area, relative to their volume, over which to take up essential materials and nutrients^37^. As such, it is expected that larger species, with a lower surface area to volume ratio, would require either a higher substrate concentration or a faster uptake rate in order to support their higher cellular requirements^37^. If higher substrate concentrations were needed then it could be expected that *S. apsteinii* might have higher CO_2_ half-saturation and optimum requirements than *E. huxleyi* and *G. oceanica* for all rates. However, *S. apsteinii* had a similar ${\rm{K}}\tfrac{1}{2}{}_{{{\rm{CO}}}_{2}}{\rm{s}}{\rm{at}}$ and CO_2_ optimum as the two smaller species for growth and photosynthetic carbon fixation rates and a slightly higher CO_2_ optimum requirement for calcification rates (Table 2,^20,30^). This suggests that *S. apsteinii* would need to support its greater substrate demand by fixing more carbon per unit surface area than smaller species. To see if this was the case, daily carbon fixation per unit surface area was calculated using carbon fixation rates (POC, PIC and TPC from^16,19,20^ and this paper at 20 °C) and average cell diameters of 5.59, 9.33, and 17.59 *μ*m for *E. huxleyi*, *G. oceanica* and *S. apsteinii*, respectively.

**Figure 5:**
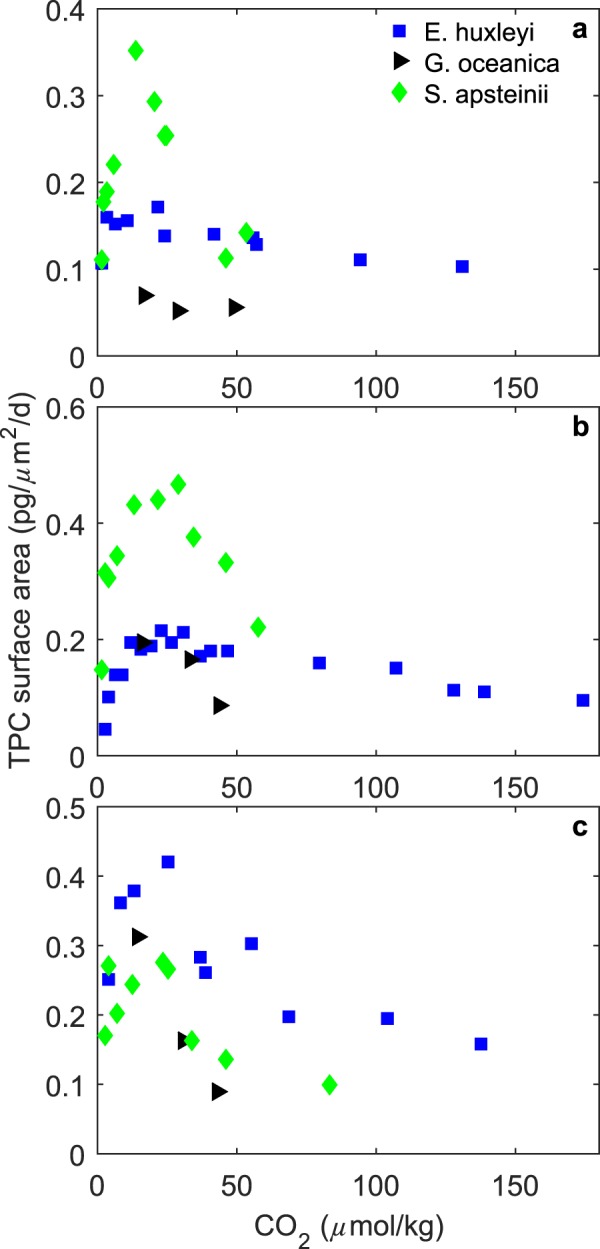
Total particulate carbon production rate per unit surface area (*μ*m^2^) for *E. huxleyi*, *G. oceanica* and *S. apsteinii* across a range of CO_2_ concentrations and at (**a**) 50, (**b**) 150–200, and (**c**) 515–600 *μ*mol photons m^−2^ s^−1^.

Based on these calculations, *S. apsteinii* fixes more carbon per unit surface area per day than either *E. huxleyi* or *G. oceanica* under most CO_2_ conditions at low to moderate light intensities (Fig. 5a and b). However, at higher light intensities it fixes approximately the same amount of carbon per unit surface area per day (Fig. 5c). The relative decrease in carbon fixation at these higher light intensities in comparison to *E. huxleyi* and *G. oceanica* is likely due to a higher light sensitivity (see below). Meanwhile below this threshold, the generally higher fixation of carbon per unit surface area per day by *S. apsteinii* can be achieved by higher inorganic carbon transporter density (higher substrate uptake), or by less diffusive CO_2_ leakage from the cell. It may be that by having a proportionally lower amount of the cells internal volume interacting with the surrounding media (due to a much lower surface area to volume ratio) *S. apsteinii* loses proportionally less CO_2_ through leakage. While decreased leakage in larger coccolithophore species has been suggested previously^37^, this difference in leakage was thought to be driven by the uptake of different proportions of CO_2_ and ${{\rm{HCO}}}_{3}^{-}$ between the species rather than size alone. While evidence suggests that carbon uptake efficiency is higher in some species and phytoplankton groups (i.e.^38,39^), it is not yet clear if cell size has a consistent effect on carbon uptake efficiencies.

Sensitivity to high CO_2_/low pH varied between the three rates with calcification being more sensitive than photosynthesis and growth (Table 2). This agrees with previous work on *G. oceanica*, *E. huxleyi* and *C. pelagicus*, and provides additional evidence for the general notion that calcification by coccolithophores will be negatively impacted by future ocean changes^16,18–20^. Sensitivity of calcification rates to high CO_2_ $({\rm{K}}\tfrac{1}{2}{}_{{{\rm{CO}}}_{2}}{\rm{i}}{\rm{nhib}})$ are similar between *S. apsteinii* and *E. huxleyi*, with *G. oceanica* being much more sensitive^20,30^. The steeper decline of calcification rates, beyond optimum CO_2_, of *G. oceanica* compared to *E. huxleyi* has previously been speculated to be connected to a higher degree of inorganic versus organic carbon production (PIC:POC) in *G. oceanica*^18,20^. The relatively greater production of intracellular H^+^, via calcification, makes it more difficult for species species with higher PIC content to maintain intracellular pH homeostasis^17^. In this regard, *S. apsteinii* with a similar PIC:POC as *E. huxleyi* would fit into this concept.

Significant inhibition of all rates by light was observed in *S. apsteinii* when CO_2_ levels exceeded 100 *μ*atm (Fig. 3, Table 3). That is in sharp contrast to rates in *E. huxleyi* and *G. oceanica* for which little light inhibition is observed regardless of *f* CO_2_ levels^19,40–45^. Optimal light intensities for growth, calcification and photosynthesis of *S. apsteinii* also differ from those in *E. huxleyi* and *G. oceanica*. While optimal physiological rates for *S. apsteinii* are observed at light intensities between ∼120–263 *μ*mol photons m^−2^ s^−1^, depending upon *f* CO_2_ level, optimal physiological rates for *G. oceanica* and *E. huxleyi*, under comparable experimental conditions, have been observed at light intensities of 400–800 *μ*mol photons m^−2^ s^−1^ and 500–1200 *μ*mol photons m^−2^ s^−1^, respectively (Table 2,^19,20^). *S. apsteinii* also tends to half-saturate its rates $({\rm{K}}\tfrac{1}{2}{}_{{\rm{PAR}}}{\rm{s}}{\rm{at}})$ at lower light intensities than either *E. huxleyi* or *G. oceanica* under most CO_2_ conditions (Table 2,^20,30^, unpublished *E. huxleyi* data). This suggests that *S. apsteinii* may be a lower light adapted species which is also supported by the fact that it has been observed to grow at light intensities as low as 5 *μ*mol photons m^−2^ s^−1^ ^46^. Oceanic abundance data for *S. apsteinii* is sparse, as a result it is currently not possible to confirm if similar low light preferences are observed in the natural environment.

**Figure 6:**
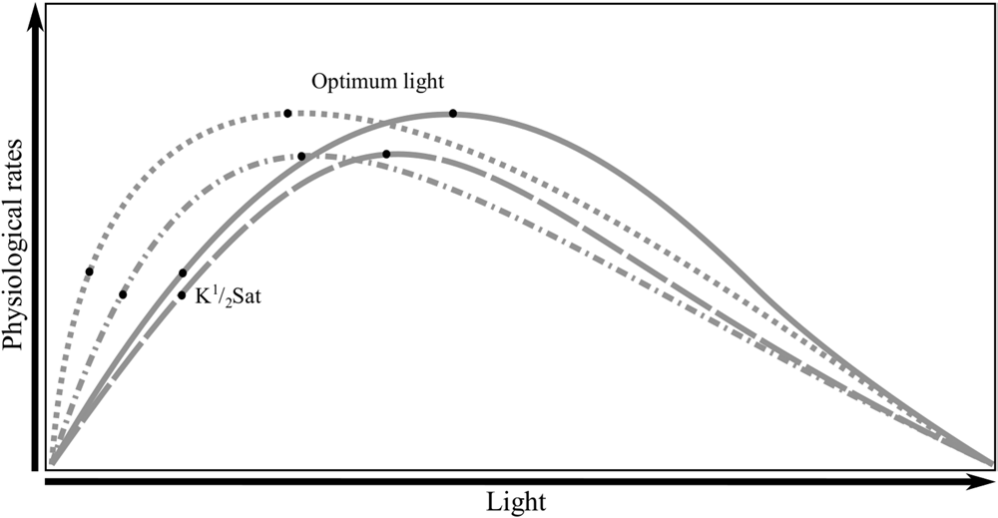
Conceptual diagram depicting how the light intensities required to support half-saturated $({\rm{K}}\tfrac{1}{2}{\rm{sat}})$ and optimum rates change with changing CO_2_. While the solid line represents the default response to rising light levels, the dotted line represents an increase in substrate uptake efficiency with rising CO_2_, the dashed line represents an increase in H^+^ inhibition with rising CO_2_ and the dot-dashed line represents an increase in both substrate uptake efficiency and H^+^ inhibition with rising CO_2_.

As *f* CO_2_ levels increased *S. apsteinii* needed lower light intensities to sustain optimal rates. These results agree with those for *G. oceanica*, which also required less light to support optimum calcification and photosynthetic carbon fixation rates as *f* CO_2_ increased^30^. The results also agree with those for *E. huxleyi* growth rates, which required less light with rising *f* CO_2_, but not calcification or photosynthetic carbon fixation rates which were insensitive to changing light intensity^20^ (Table S3). In all three-above species, the increase in *f* CO_2_ levels also resulted in an overall decrease in maximum rates^20,30^ (Table 2). Most phytoplankton acquire inorganic carbon through a combination of passive CO_2_ diffusion into the cell, and CO_2_ concentrating mechanisms (CCMs) which actively transport inorganic carbon into the cell^47,48^. As CO_2_ concentrating mechanisms are an active process, they require energy to function^39,48^. The efficiency of passive uptake depends upon the cell to seawater CO_2_ concentration gradient (uptake versus leakage rates), membrane permeability and area^39^. So, assuming that cell size and membrane permeability stay constant, increasing seawater CO_2_ concentrations increase the diffusive influx and reduce loss through leakage. This would reduce the need for an active CCM thus lowering the cell’s energy requirements. As a result, higher substrate uptake efficiency could be indicated by a decrease in light levels needed to reach half-saturated and optimal rates (see Fig. 6 dotted line). Another explanation, for reduced light requirements could be the inhibiting effects of high CO_2_/H^+^. As CO_2_/H^+^ concentrations increase to above optimum levels it suppresses maximum rates to lower and lower absolute rates. Lower rates result in decreased demand for resources such as carbon. This lower substrate demand could result in decreased CCM activity thereby decreasing optimum light requirements under elevated CO_2_/H^+^ (Fig. 6 dashed line). All three species show decreased maximum rates under elevated CO_2_/H^+^. As a result, decreased energy requirements because of inhibited maximum rates could explain decreased optimum light requirements in *E. huxleyi*, *G. oceanica* and *S. apsteinii*. However, *G. oceanica* also shows indications of increased substrate uptake efficiency, through decreasing ${\rm{K}}\tfrac{1}{2}{}_{{\rm{PAR}}}{\rm{s}}{\rm{at}}$ and optimal light requirements^30^. So, for *G. oceanica* it appears that increasing CO_2_/H^+^ may decrease light optimal requirements through a combination of increased substrate uptake efficiency and through inhibition of maximum rates (Fig. 6 dot-dashed line).

As well as decreasing light intensities for optimal rates, increasing *f* CO_2_ also resulted in a narrowing of the light tolerances (light niche) of *S. apsteinii*. This was driven by a combined increase in light intensities required for half saturation of rates, and a decrease in light intensities required to half inhibit rates with rising *f* CO_2_ (Table 3). The CO_2_ niche of this species was also observed to narrow with increasing light intensity, with an increased sensitivity to high CO_2_ and a decrease in CO_2_ requirements for optimal physiological rates (Table 2). So *S. apsteinii*, when already under stress from one environmental condition, becomes less tolerant to other extreme environmental conditions, similar to *E. huxleyi* and *G. oceanica* for high CO_2_ sensitivity under rising temperature^20,30^. However, when exposed to the same stress of rising light intensities, *E. huxleyi* becomes less sensitive to high CO_2_ conditions, while the sensitivity of *G. oceanica* to high CO_2_ depends upon the rate considered^20,30^. Similarly, when exposed to the stress of rising CO_2_ conditions, neither *E. huxleyi* or *G. oceanica* show any change in sensitivity to high light conditions^20,30^ (Table S3). So, it would appear that the response to multiple environmental stressors is species-specific. This adds weight to the suggestion that coccolithophore assemblages may undergo shifts in species composition, under future ocean change.

Depending on the emission scenario, *p* CO_2_ levels are projected to reach between 420 to 985 *μ*atm by 2100 resulting in ocean temperature increases of 2.6 to 4.8 °C (RCP 2.6–8.5)^49,50^. Ocean warming is expected to strengthen stratification of the water column, resulting in a shallower mixed layer and thus a higher average light availability within the mixed layer^2,51^. The dependence of light responses on *f* CO_2_ levels, and vice-versa, could have important implications for *S. apsteinii* under future ocean conditions where both light and *f* CO_2_ availabilities are expected to change. *S. apsteinii* already appears to be a low light adapted species with rates saturating at relatively low light intensities under current day *f* CO_2_ conditions (<170 *μ*mol photons m^−2^ s^−1^). Considering that light levels to saturate and inhibit rates for this species increase and decrease, respectively, with rising *f* CO_2_ (Table 3), this species could become restricted to a narrower light range under predicted future ocean conditions.

## Conclusion

*S. apsteinii* appears to be a low light adapted species, with a similar optimum curve response to rising CO_2_ as *E. huxleyi* and *G. oceanica*, *C. pelagicus* and *C. leptoporus*. Calculations suggest that for *S. apsteinii*, a single unfavourable growth condition (CO_2_, or light) will result in increased sensitivity to changes in other environmental variables. This contrasts with *E. huxleyi*, which either becomes less sensitive or shows no change in sensitivity to changes in other environmental variables when at extreme CO_2_ or light conditions, and *G. oceanica* whose change in sensitivity varies between different physiological rates. With light and CO_2_ levels both set to increase over the coming century^2,51^ *S. apsteinii* could become restricted to narrower light and CO_2_ ranges while under rising temperature, *E. huxleyi* and *G. oceanica* could become more sensitive to high CO_2_. These species-specific changes in temperature/light/CO_2_ sensitivity are likely to affect coccolithophore community composition under future ocean conditions.

Data from *E. huxleyi*, *G. oceanica* and *S. apsteinii* indicate that rising CO_2_ levels will also result in decreased optimum light requirements of all species as a result of reduced maximum rates and, in the case of *G. oceanica*, increased substrate uptake efficiency. This adds further evidence to the idea that rising CO_2_ will not only result in changes in species composition, but also in community-wide shifts in total organic and inorganic carbon production by coccolithophores, with consequent effects on local carbon cycling and sequestration.

## Methods

### Experimental set-up

Mono-specific cultures of *S. apsteinii* (strain RCC1456 isolated from the Mediterranean Sea, Spain) were grown in artificial seawater (ASW)^52^ at a salinity of 35 and temperature of 20 °C across a *p* CO_2_ gradient of ∼50–7000 *μ*atm. Cultures were incubated at 50, 100, 200 and 515 *μ*mol photons m^−2^ s^−1^ of photosynthetically active radiation (PAR) on a 16:8 h light-dark cycle in a Panasonic Versatile Environmental Test Chamber (MLR-352-PE). Light intensities, for bottle placement, within the chamber were measured using a LI-193 spherical sensor (LI-COR). Cells were pre-acclimated to experimental conditions for 7–13 days depending upon cell division rates. Initial cell densities for each bottle varied between 35–50 cells ml^−1^ depending upon the treatment conditions.

### Media preparation

Artificial seawater (ASW), salinity 35, was prepared according to^52^, minus the initial addition of bicarbonate. The ASW was enriched with f/8 trace metals and vitamins^53^, 64 *μ*mol kg^−1^ nitrate (${{\rm{NO}}}_{3}^{-}$), 4 *μ*mol kg^−1^ phosphate (${{\rm{PO}}}_{4}^{3-}$), 10 nmol kg^−1^ SeO_2_ and 1 ml kg^−1^ of sterile filtered seawater collected from Shelly beach, Ballina. ASW medium was sterile-filtered (0.2 *μ*m pore size, Whatman^TM^ Polycap 75 AS) into autoclaved polycarbonate bottles, Nalgene for acclimation (0.5 L) or experimentation (2 L), with a small head-space left for the adjustment of carbonate chemistry conditions.

### Carbonate chemistry manipulation

Total alkalinity (TA) and dissolved inorganic carbon (DIC) for each treatment was adjusted through calculated additions of hydrochloric acid (certified 3.571 mol L^−1^ HCl, Merck) and 1.4 mol L^−1^ sodium carbonate (Na_2_ CO_3_ Sigma-Aldrich, TraceSELECT quality dried for 2 hours at 240 °C). At the end of the experiment samples for TA and DIC were taken, stored and measured following methods used in^20^. Carbonate chemistry speciation (${{\rm{HCO}}}_{3}^{-}$, ${{\rm{CO}}}_{3}^{2-}$, CO_2_, pH) for each treatment was calculated from measured TA, DIC, temperature, salinity and [${{\rm{PO}}}_{4}^{3-}$] using the program CO2SYS^54^, the stoichiometric equilibrium constants (K_1_ and K_2_) for carbonic acid determined by^55^ and refitted by^56^, K_S_ for sulphuric acid determined by^57^ and K_B_ for boric acid following^58^.

### Cell densities and particulate and dissolved carbon

Cell densities for each treatment were checked every 2–3 days using a flow cytometer (Becton Dickinson FACSCalibur). Living cells were detected by scatter plots of red autofluorescence in relation to orange fluorescence of the cells (FL3 vs. FL2). Specific growth rate (*μ*) was determined using cell density counts from the beginning and at the end of the experiment. Growth rate was calculated as:

1 $$\mu =\frac{\mathrm{ln}({{\rm{C}}}_{{\rm{t}}})-\,\mathrm{ln}({{\rm{C}}}_{{\rm{0}}})}{{\rm{d}}}$$

where C_t_ and C_0_ are the cell densities at the end and the beginning of the experiment respectively and d is incubation length in days. Calcification and photosynthetic rates were obtained by multiplying cellular quotas of particulate inorganic (calcification) or particulate organic (photosynthesis) carbon with growth rates. End of experiment sampling started approximately two hours after the onset of the light period and lasted no longer than 3 hours. Duplicate samples for total and organic particulate carbon (TPC and POC) were filtered (−200 mbar) onto pre-combusted (500 °C for 4 hours) Whatmann GF/F filters and stored in pre-combusted (500 °C for 4 hours) glass petri-dishes at −20 °C. Prior to analysis, POC filters were treated with 37% fuming HCl in a desiccator for 2 hours to remove all particulate inorganic carbon (PIC). TPC and POC filters were dried and analysed for carbon content and carbon isotopic signatures on an elemental analyser (Flash EA, Thermo Fisher) coupled to an isotope ratio mass spectrometer (IRMS, Delta V plus, Thermo Fisher) according to^59^. PIC content was calculated by subtracting measured values of particulate organic carbon (POC) from total particulate carbon (TPC).

### Data fitting

Cellular metabolic rates (n = 39 for calcification and photosynthesis and n = 37 for growth) were fitted to a non-linear equation derived in^30^ as

2 $${\rm{MR}}({S},{I},{H})=\frac{{{\rm{k}}}_{1}{\rm{SI}}}{{{\rm{k}}}_{{\rm{2}}}{\rm{H}}+{{\rm{k}}}_{{\rm{3}}}{\rm{SH}}+{{\rm{k}}}_{{\rm{4}}}{\rm{I}}+{{\rm{k}}}_{{\rm{5}}}{\rm{SI}}+{{\rm{k}}}_{{\rm{6}}}{{\rm{SHI}}}^{{\rm{2}}}}$$

where the metabolic rate (MR) of photosynthesis, calcification or growth is dependent on substrate (S = CO_2_ and ${{\rm{HCO}}}_{3}^{-}$), [H^+^] (H) and light (I) conditions, and fit coefficients k_1_, k_2_, k_3_, k_4_, k_5_ and k_6_. For the experiment, a higher number of treatment levels was used at the expense of replication within treatments. This approach provides more information on the functional relationship, and tipping points, between carbonate chemistry and cellular rates without a significant loss of statistical power (see^60^). Statistical results (R^2^, fit coefficients, p-values, F-values and degrees of freedom), as well as V_max_ (maximum production rates), ${\rm{K}}\tfrac{1}{2}{}_{{{\rm{CO}}}_{2}}{\rm{i}}{\rm{nhib}}$ (conditions under which rates are reduced to half of maximum by either high light or high CO_2_ concentrations), ${\rm{K}}\tfrac{1}{2}{}_{{{\rm{CO}}}_{2}}{\rm{s}}{\rm{at}}$ (conditions where low light or low CO_2_ availability reduces rates to half of maximum) and optima (CO_2_ or light conditions where V_max_ is reached), are presented in Tables 1, 2 and 3, respectively. Please note changes in CO_2_ optima and half-saturation of less than 10 *μ*mol kg^−1^ were considered to be within the uncertainties of the model fit.

### Coccolith morphology

*S. apsteinii* is unusual in that it bears two types of coccoliths; the plate-like muroliths and the vase-shaped lopadoliths^61^. To assess potential changes in coccolith morphology under different treatments, samples for scanning electron microscopy (SEM) were filtered onto Nucleopore^TM^ polycarbonate membrane filters (25 mm diameter, 0.8 *μ*m pore size) and air-dried at room temperature over 12 hours. Samples were then stored in a desiccator until analysis. Samples were mounted onto metallic stubs using sticky tabs and sputter-coated with gold before being visualized using a ZEISS EVO/LS15 scanning electron microscope. For each treatment 80–100 cells were examined and the number of muroliths and lopadoliths per cell, average of murolith and lopadolith length and width per cell, and the ratio of muroliths to lopadoliths were recorded. Due to time constraints, only a sub-set of treatments could be examined. These were; 100, 400 and 2000 *μ*atm *f* CO_2_ at 50 and 515 *μ*mol photons m^−2^ s^−1^ and 100, 200, 400 and 2000 *μ*atm at 100 *μ*mol photons m^−2^ s^−1^ of PAR. Comparisons between treatments were made using a Kruskal-Wallis ANOVA with an alpha level of significance of 0.05. The Kruskall-Wallis ANOVA was chosen firstly to avoid an inflated Type I error rate which can occur if making multiple comparisons between groups, and secondly to account for the slight non-normal distribution of the SEM data. The mass of PIC per coccolith was calculated by dividing PIC quota per cell by the total number of liths (muroliths + lopadoliths) for each treatment. Patterns in PIC per coccolith were tested using a linear regression with an alpha of significance of 0.05. Linear regressions were chosen for the reasons explained in Section ‘Cell densities and particulate and dissolved carbon’ above.

## Supplementary information

Supplementary material for: The innfluence of light and carbonate chemistry on metabolic rates in Scyphosphaera apsteinii: A discussion of species-specific sensitivities and requirements

## Data Availability

Datasets on physiological rate responses generated and compared during the current study are available in this published article (and its Supplementary Information files) and in^20,30^. Datasets containing the raw scanning electron microscopy values are available from the corresponding author on reasonable request.
