# Supplementary material for: A comparison of species specific sensitivities to changing light and carbonate chemistry in calcifying marine phytoplankton
Source: Sci Rep. 2019 Feb 21;9:2486. doi: 10.1038/s41598-019-38661-0 (PMC6385225; doi:10.1038/s41598-019-38661-0)
Supplement: Supplementary file 1 — Supplementary material for: The innfluence of light and carbonate chemistry on metabolic rates in Scyphosphaera apsteinii: A discussion of species-specific sensitivities and requirements [file 41598_2019_38661_MOESM1_ESM.pdf]

Supplementary material for: A comparison of species specific sensitivities to changing light and carbonate chemistry in calcifying marine phytoplankton

Natasha A. Gafar*, Bradley D. Eyre & Kai G. Schulz

15th December 2018

*Corresponding author: n.gafar.10@student.scu.edu.au

Discussion: Coccolith geometry

Measurements of cell volume, lopadolith and murolith size and number were taken for a subset of light and $f\mathrm{CO_2}$ treatments. While cell volume did appear to show slight differences with changing $f\mathrm{CO_2}$ at different light intensities, there was no consistent trend (Table S2). The length and width of both muroliths and lopadoliths varied greatly both within and between treatments (Table S2). As a result, clear changes in response to changing light intensity and $f\mathrm{CO_2}$ were difficult to distinguish. One interesting trend observed was when average murolith lengths increased with changing $f\mathrm{CO_2}$ and light intensity so would the average width. As a result, the shape of muroliths did not change with changing light or $f\mathrm{CO_2}$. In contrast, the length and width of lopadoliths followed different patterns of response under changing $f\mathrm{CO_2}$ and changing light. It should be noted that *S. apsteinii* has previously been observed to have lopadoliths of a variety of shapes and sizes on a single cell even under stable environmental conditions [1]. This would explain the large amount of variability seen within each treatment in terms of lopadolith size. Having a wide range of lopadolith sizes even under constant conditions makes identifying a treatment effect difficult.

With increasing light intensity, the murolith to lopadolith ratio increased from ∼six to seven at 100 μatm $\mathrm{CO_2}$ and from ∼six to eight at 400 μatm $\mathrm{CO_2}$ (Table S2). This agrees with [2] who observed an increase in the ratio of muroliths to lopadoliths from 5 to 100 $\mu\mathrm{mol\,photons\,m^{-2}s^{-1}}$. While the change in ratio is smaller than that observed in [2], this may be a result of different light ranges used between the studies. The largest change in murolith to lopadolith ratio in [2] was observed between 5 and 30 $\mu\mathrm{mol\,photons\,m^{-2}s^{-1}}$ which is much lower than our lowest light treatment of 50 $\mu\mathrm{mol\,photons\,m^{-2}s^{-1}}$. So, it may be that the largest changes in lith ratio occur only under very low/limiting light conditions after which the ratio changes more slowly with changes in light intensity. Alternatively, different culture conditions in terms of light:dark cycle, nutrient concentration, nutrient composition and temperature may have influenced ratios.

S. apsteinii contained more PIC per average lith (mix of both types) in treatments with higher calcification rates (Fig. 4). This was not connected to changes in the cellular abundance of lopadoliths or muroliths as there was no significant trend of calcification rate with murolith number per cell, lopadolith number per cell or murolith to lopadolith ratio. There were some changes in lith length and width, i.e. there tends to be slightly larger lopadoliths at 400μatm, however, they were not significant due to the large within-treatment variation. Changes in lith size appear mostly independent of changes in calcification rate (i.e. liths are not always bigger at higher calcification rates see Table S2). Based on this, the increasing PIC weight of the liths, with increasing calcification rate, is possibly due to increased lith thickness. Increases in lith weight with rising calcification rate have been observed for *E. huxleyi* [3]. Interestingly, however, increases in lith weight in *E. huxleyi* were associated with increasing size, suggesting different underlying impacts of calcification rate on $\mathrm{CaCO_3}$ crystal growth in the two species.

Tables

Table S1: Carbonate chemistry speciation, temperature, light conditions and measured physiological parameters for *S. apsteinii* recorded in this study. Note pH is recorded on the total scale.

μ (d^{-1})	POC Rate ($\frac{pg\ C}{cell\ d}$)	PIC Rate	POC Quota ($\frac{pg\ C}{cell\ d}$)	PIC Quota	PIC:POC	fCO_2 (μatm)	$[CO_2]$ ($\frac{\mu mol}{kg}$)	$[HCO_3^-]$	$[CO_3^{2-}]$	pH_T	$[H^+]$ ($\frac{mol}{kg}$)	TA ($\frac{\mu mol}{kg}$)	DIC	Light ($\frac{\mu mol}{m^2\ s}$)
0.137	60.53	47.88	441.83	349.50	0.79	47	1.52	1064.6	520.5	8.736	1.84E-09	2358.83	1586.61	50
0.245	96.41	75.23	393.49	307.08	0.78	69	2.23	1207.2	457.0	8.625	2.37E-09	2343.06	1666.47	50
0.262	101.98	81.70	389.22	311.82	0.80	94	3.06	1349.0	416.2	8.536	2.91E-09	2378.88	1768.23	50
0.257	94.28	121.11	366.92	471.35	1.28	183	5.94	1601.9	302.2	8.322	4.76E-09	2350.66	1909.99	50
0.307	140.13	202.17	456.45	658.52	1.44	426	13.79	1865.9	176.6	8.023	9.49E-09	2305.99	2056.31	50
0.335	129.61	156.05	386.89	465.83	1.20	623	20.19	1947.3	131.4	7.876	1.33E-08	2276.37	2098.90	50
0.332	109.41	136.62	329.55	411.52	1.25	766	24.82	2059.7	119.6	7.810	1.55E-08	2357.44	2204.10	50
0.266	126.55	119.50	475.75	449.24	0.94	735	23.84	2096.1	128.9	7.836	1.46E-08	2415.42	2248.87	50
0.176	64.49	46.05	366.43	261.66	0.71	1425	46.18	2217.9	74.5	7.573	2.67E-08	2404.15	2338.64	50
0.146	72.12	65.24	492.42	445.44	0.90	1648	53.42	2215.6	64.3	7.509	3.1E-08	2377.12	2333.36	50
0.273	116.64	92.60	427.25	339.20	0.79	87	2.83	1302.2	419.5	8.555	2.79E-09	2343.66	1724.46	100
0.395	143.10	135.08	362.28	341.98	0.94	125	4.06	1449.0	361.9	8.444	3.6E-09	2346.60	1815.00	100
0.451	185.80	170.14	411.98	377.25	0.92	234	7.57	1686.6	262.8	8.239	5.76E-09	2338.63	1956.99	100
0.562	274.64	NaN	488.68	NaN	NaN	446	14.45	1895.2	173.8	8.009	9.79E-09	2327.56	2083.43	100
0.566	243.71	234.42	430.59	414.16	0.96	762	24.70	2059.5	120.1	7.812	1.54E-08	2358.65	2204.39	100
0.503	228.97	229.38	455.20	456.03	1.00	1171	37.96	2129.3	83.6	7.640	2.29E-08	2338.78	2250.86	100
0.385	174.37	165.94	452.90	431.02	0.95	1627	52.74	2165.3	62.2	7.505	3.13E-08	2322.34	2280.27	100
0.345	176.89	NaN	512.72	NaN	NaN	1967	63.75	2195.4	52.9	7.428	3.73E-08	2329.37	2312.02	100
0.262	125.15	68.92	477.66	263.06	0.55	2152	69.75	2216.9	49.3	7.394	4.04E-08	2341.87	2335.93	100
0.199	161.41	91.13	811.09	457.93	0.56	2078	67.34	2247.5	52.5	7.415	3.85E-08	2379.96	2367.35	100
0.283	95.13	48.26	335.88	170.40	0.51	54	1.75	1113.2	495.9	8.695	2.02E-09	2346.76	1610.92	200
0.344	190.79	114.96	554.21	333.92	0.60	78	2.52	1251.5	434.9	8.587	2.59E-09	2332.71	1688.85	200
0.419	155.53	140.62	370.84	335.27	0.90	121	3.92	1431.5	365.5	8.454	3.52E-09	2338.55	1800.88	200
0.381	197.72	136.20	518.53	357.19	0.69	223	7.23	1667.8	269.3	8.255	5.56E-09	2335.98	1944.29	200
0.371	238.41	180.35	643.17	486.54	0.76	410	13.28	1862.0	182.6	8.038	9.16E-09	2316.46	2057.86	200
0.393	208.56	218.31	530.83	555.66	1.05	672	21.77	2039.9	133.7	7.863	1.37E-08	2372.01	2195.36	200
0.365	247.97	206.75	679.16	566.26	0.83	893	28.96	2112.4	107.8	7.754	1.76E-08	2380.66	2249.15	200
0.346	200.29	166.09	578.14	479.41	0.83	1058	34.27	2174.8	96.5	7.694	2.02E-08	2414.80	2305.63	200
0.336	187.64	135.11	558.46	402.11	0.72	1423	46.11	2206.2	73.9	7.571	2.68E-08	2391.00	2326.19	200
0.316	134.86	81.36	426.16	257.11	0.60	1780	57.70	2272.6	62.6	7.487	3.26E-08	2429.39	2392.95	200
0.278	95.93	68.92	344.80	247.74	0.72	79	2.56	1273.8	443.9	8.589	2.58E-09	2373.44	1720.25	515
0.296	156.46	107.22	527.80	361.70	0.69	119	3.85	1435.3	374.3	8.463	3.45E-09	2362.27	1813.43	515
0.300	119.11	77.83	396.81	259.27	0.65	210	6.79	1669.0	286.8	8.282	5.23E-09	2378.08	1962.63	515
0.239	133.79	103.81	559.94	434.45	0.78	387	12.55	1876.5	196.3	8.066	8.59E-09	2363.14	2085.42	515
0.318	145.48	121.59	458.06	382.81	0.84	722	23.38	2048.8	125.6	7.834	1.47E-08	2361.14	2197.71	515
0.244	134.04	123.90	550.41	508.76	0.92	781	25.30	2114.8	123.6	7.813	1.54E-08	2420.96	2263.70	515
0.228	101.71	57.43	446.31	251.98	0.56	1041	33.73	2178.1	98.4	7.701	1.99E-08	2422.48	2310.23	515
0.214	67.93	64.78	316.85	302.15	0.95	1414	45.83	2177.8	72.4	7.568	2.7E-08	2359.42	2296.02	515
0.160	67.54	28.17	422.83	176.38	0.42	2569	83.27	2397.4	48.3	7.351	4.46E-08	2518.28	2528.92	515

Table S2: Coccolith (murolith and lopadolith) morphology, cell volume and total whole and burst cells observed under different light intensities and CO_2 treatments from the present study. Values in brackets represent the standard deviation of all measured cells within a treatment.

Light (PAR)	CO_2 (μatm)	Number Muroliths	Murolith length (μm)	Murolith width (μm)	Number Lopadoliths	Lopadolith length (μm)	Lopadolith width (μm)	Murolith-Lopadolith ratio	Cell volume (μm^3)
50	100	11.27 (3.0)	8.13 (0.54)	5.9 (0.45)	2.34 (0.98)	7.92 (1.3)	9.81 (1.1)	5.71 (3.2)	2378 (678)
	400	12.53 (2.9)	8.19 (0.64)	5.87 (0.53)	2.32 (0.82)	10.0 (1.8)	10.79 (1.4)	6.0 (2.4)	2473 (799)
	2000	12.85 (2.8)	7.57 (0.47)	5.68 (0.38)	2.22 (1.0)	6.3 (1.3)	8.18 (1.1)	7.09 (4.2)	2218 (922)
100	100	12.16 (2.9)	8.48 (0.56)	6.11 (0.46)	2.34 (0.88)	8.05 (1.2)	11.25 (1.1)	6.01 (3.0)	2424 (708)
	200	10.82 (2.7)	8.91 (0.71)	6.39 (0.63)	2.01 (0.86)	8.33 (1.8)	11.86 (1.5)	6.35 (3.3)	3109 (913)
	400	11.86 (2.7)	9.1 (0.54)	6.6 (0.51)	2.05 (0.78)	9.98 (1.4)	12.65 (1.2)	6.51 (3.0)	2634 (682)
	2000	10.57 (2.8)	8.56 (0.62)	6.46 (0.47)	2.56 (1.0)	7.08 (1.7)	10.46 (1.3)	4.59 (2.1)	4031 (1333)
515	100	13.87 (2.9)	8.51 (0.65)	6.19 (0.58)	2.39 (0.92)	7.74 (1.3)	10.6 (0.98)	6.81 (3.3)	3411 (802)
	400	13.5 (2.9)	8.3 (0.58)	6.0 (0.51)	2.01 (0.89)	8.38 (2.1)	11.09 (1.8)	7.83 (4.2)	3013 (550)
	2000	14.22 (3.3)	7.96 (0.46)	5.93 (0.38)	2.81 (1.0)	7.9 (1.6)	9.01 (0.85)	5.92 (3.2)	2818 (111)

Table S3: Calculated optimum light intensities, maximum rates (V_{max}) and light $K_{\frac{1}{2}}$ values of *E. huxleyi* at 20°C and 132, 370 and 1200 μatm using the fit equation and fit coefficients from [4].

**Light**	**132 μatm**	**370 μatm**	**1200 μatm**
**Optima (μmol photons $m^{-2}s^{-1}$)**			
Calcification	2500	2500	2500
Photosynthesis	2500	2500	2500
Growth rate	2097	1238	764
**V_{max} (pg C cell^{-1} d^{-1} or d^{-1})**			
Calcification	9.6	14.9	14.4
Photosynthesis	13.3	23.3	42.4
Growth rate	1.18	1.55	1.52
**$K_{\frac{1}{2}PAR}$inhib (μmol photons m^{-2} s^{-1})**			
Calcification	> 2500	> 2500	> 2500
Photosynthesis	> 2500	> 2500	> 2500
Growth rate	> 2500	> 2500	> 2500
**$K_{\frac{1}{2}PAR}$sat (μmol photons m^{-2} s^{-1})**			
Calcification	9.3	51.5	167.5
Photosynthesis	20.9	110.7	739.2
Growth rate	3.4	8.5	17.9

References

1. Siesser, W. G. Calcareous nannofossil genus Scyphosphaera: Structure, taxonomy, biostratigraphy, and phylogeny. *Micropaleontology* **44,** 351–384. ISSN: 0026-2803 (1998).

2. Drescher, B., Dillaman, R. M. & Taylor, A. R. Coccolithogenesis in *Scyphosphaera apsteinii* (Prymnesiophyceae). *Journal of Phycology* **48,** 1343–1361. ISSN: 1529-8817 (2012).

3. Bach, L. T., Bauke, C., Meier, K., Riebesell, U. & Schulz, K. G. Influence of changing carbonate chemistry on morphology and weight of coccoliths formed by *Emiliania huxleyi*. *Biogeosciences (BG)* **9,** 3449–3463. ISSN: 1726-4170 (2012).

4. Gafar, N. A., Schulz, K. G. & Eyre, B. D. A three-dimensional niche comparison of *Emiliania huxleyi* and *Gephyrocapsa oceanica*: Reconciling observations with projections. *Biogeosciences* **15,** 3541–3560 (2018).
